# High volume fly ash mortar containing nano-calcium carbonate as a sustainable cementitious material: microstructure and strength development

**DOI:** 10.1038/s41598-018-34851-4

**Published:** 2018-11-06

**Authors:** Huashan Yang, Yujun Che, Faguang Leng

**Affiliations:** 1 School of Materials and Architecture Engineering, Guizhou Normal University, Guiyang, China; 2 China Academy of Building Research, Beijing, 100013 China

## Abstract

The mechanisms underlying the effects of nano-calcium carbonate (NC) on the strength of high volume fly ash (FA) mortar are discussed. Two NCs are used as 2%, 4%, 6%, and 8% by weight of cementitious materials. Hydrated products of fly ash mortar containing NC was investigated using X-ray diffraction (XRD), scanning electron microscopy (SEM), thermogravimetric analysis (TG) and differential thermal gravity (DTG) analysis. Results indicate that NC could improve strength of FA mortar due to the more rapid growth of hydrated products induced by NC through additional nucleation sites. Corresponding to the highest measured strength of FA mortar, the optimal contents of NC are around 2%. In addition, the presence of 2% NC improved the microstructure of FA mortar after 180 days due to the formation of calcium carbonaluminate hydrate.

## Introduction

The environmental impact of using ordinary Portland cement (PC) to produce concrete is primarily due to the approximately 7% of carbon dioxide emission^1–4^. An overwhelming consensus has been reached on the use of mineral admixtures, such as FA, limestone powder, slag, and silica fume to replace the bulk portion of cement in concrete to reduce the carbon footprint of the concrete industry. These mineral admixtures can improve both the mechanical properties and durability of cementitious materials at later ages^5–8^.

One of such mineral admixture is FA, a by-product of coal-fired power station that has been used in cement concrete for many years. FA can improve the rheological performances of fresh concrete. Furthermore, due to the presence of silicon oxide and aluminum oxide, FA can react with CH to form C-S-H and C-A-H. These additional hydrated products fill the pores and form a denser matrix, and thus improve the strength and durability of concrete.

However, above a threshold substitution about 30%, it will adversely affect the early mechanical properties of concrete^9^. Since concrete containing high content of FA becomes popular, it is essential to compensate for the slower initial strength development. Nanoparticles, such as titanium dioxide (TiO_2_), zinc dioxide (ZnO_2_), carbon nanotubes (CNTs), nanosilica, and nanoclays, have recently been applied to improve the early strength development of concrete^10,11^. However, the production of these materials is energy intensive and therefore has direct economic costs and high environmental impacts^12^.

With increasing emphasis on the environment, it is important to investigate the relatively less energy-intensive and more environmental friendly nanoparticles. It is pointed out that NC can compensate for the adverse effects of FA on the early-age performances of concrete, and promote the development of a more environmentally friendly, lower energy-intensive and cost-benefit ratio FA concrete^13^. Thus, due to the recent innovations in nanotechnology, cementitious materials with NC have generated much research interest^14–18^.

Calcium carbonate has two functions, one as an inert filler, the other as a nucleation substrate for hydration products^19^. Through seeding effects, calcium carbonate can accelerate the rate of hydration, thus improve the early-age mechanical performances of concrete containing FA^11^. It is suggested that the additional surface provided by the calcium carbonate for the nucleation and growth of the hydration products is the cause for the observed acceleration^20^. From a chemical point of view, calcium carbonate seems to favor crystallization of monocarbonate rather than monosulfate^21^. In addition, it also reacts with tricalcium silicate and leads to calcium carbosilicate hydrate^22^. As a consequence, the presence of NC has a significant effect on the hydration of cement, which may accelerate early-age strength development, thus offset the negative effects of FA on early-age performances of FA concrete^23,24^. It is found that the short-term strength of FA concrete is improved by the addition of NC^25^.

However, there are few related studies, and those focus mostly on the early-age performances of concrete containing FA. It is a fact that NC still represents a relatively new material for application in FA mortar, and the investigation of the effects of NC on microstructure and strength development of FA mortar is insufficient. Therefore, more investigation is needed to further understand the mechanism of effects of NC on concrete, particularly in FA mortar at later ages.

The aim of this study is to understand the mechanisms by which NC affects the performances of FA mortar. In addition, the microstructure of FA mortar containing NC was investigated using SEM, XRD, TG/DTG analysis.

## Materials and Methods

### Materials

Portland cement (P.I 42.5), FA, silica sand, tap water, high range water reducer admixture (HRWRA), and two NCs (NC1 and NC2) obtained from different sources were used in this study. NC1 and NC2 were supplied by Cheng Du Micxy Chemical Co., Ltd. of China and Shanghai Yuanjiang Chemical Co., Ltd. of China, respectively. The chemical compositions of PC, FA, NC1, and NC2 are provided in Table 1. NC may well be difficult to disperse in mortar, which could be harmful to the strength development of mortar. Therefore, dispersion of NC in FA mortar is a big challenge, which requires more attention. HRWRA is the most used dispersing agents in the application of nanoparticles. Therefore, in order to obtain the best dispersion state of the NC in the FA mortar, utilizing a HRWRA would be necessary.

**Table 1:** Chemical composition and physical properties of cementitious materials.

Items	Chemical composition (%)			
	PC	FA	NC1	NC2
SiO_2_	21.5	45.3	0.6	0.5
Al_2_O_3_	5.1	22.1	0.3	0.1
Fe_2_O_3_	3.3	12.4	0.1	0.1
CaO	62.9	3.4	54.4	55.6
MgO	2.2	0.9	0.5	0.3
SO_3_	2.5	2.9	—	0.1
CO_2_	1.3	4.9	43.7	43.2
Others	1.2	8.1	0.4	0.1

**Figure 1:**
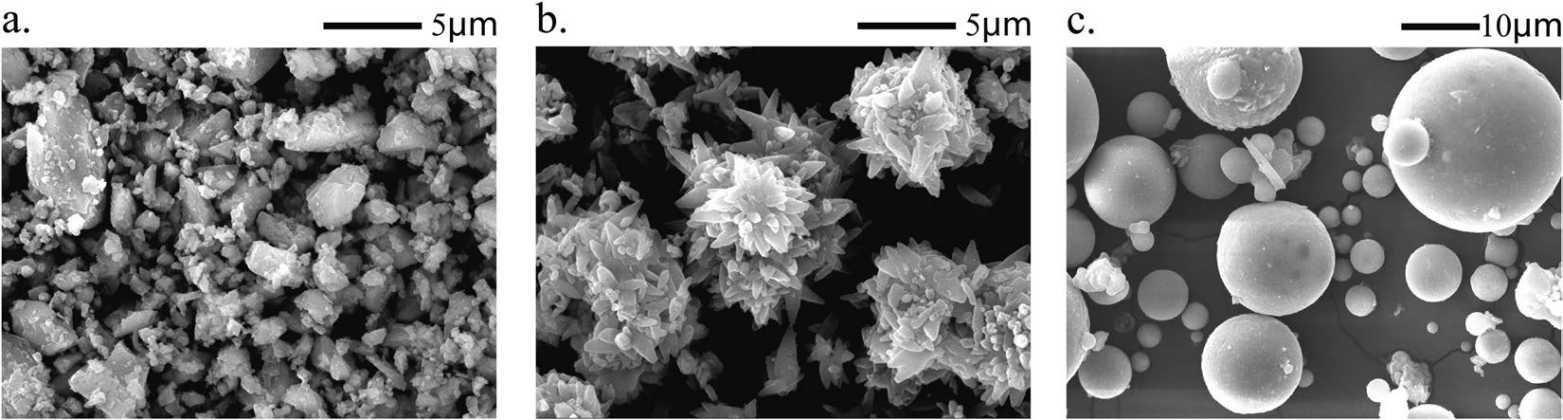
SEM images of materials ((**a**) NC1; (**b**) NC2; and (**c**) FA).

**Figure 2:**
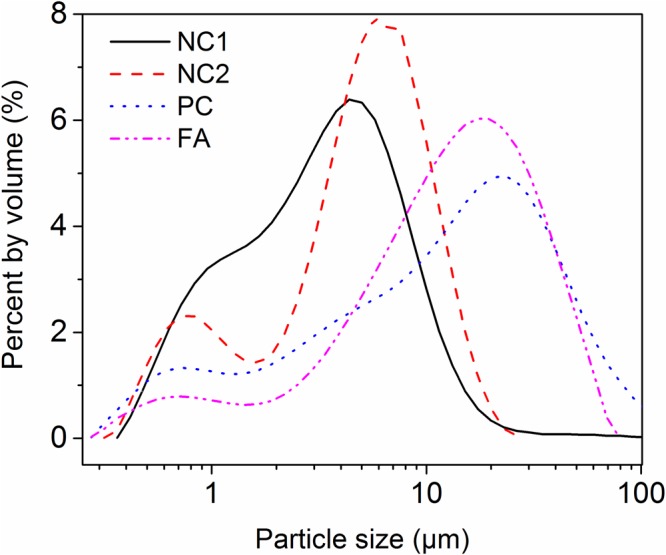
Particle size distributions of materials.

**Figure 3:**
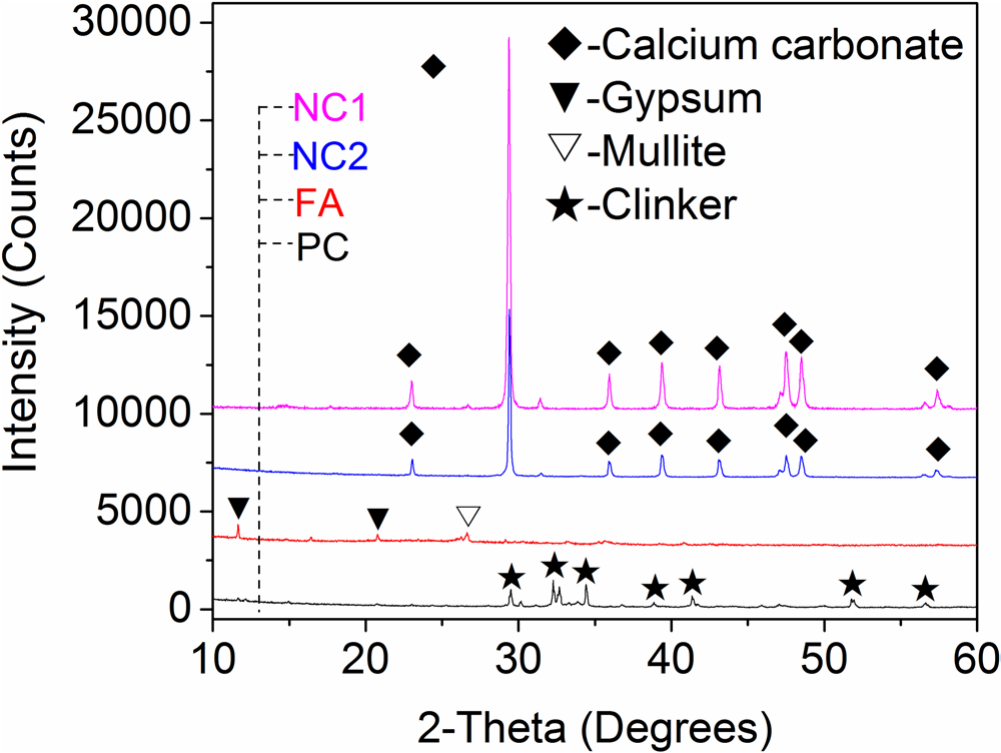
XRD analysis of materials.

The SEM image of NC1, see Fig. 1(a), indicates that most of the particles are in a better dispersed state, although there are some agglomerated particles. The difference is probably the result of milling conditions^26^ and source rock properties. The most particles of FA are round, as showed in Fig. 1(c). It is helpful to increase the flowability of mortar. Particle size distributions (PSDs) of the NC1, NC2, PC, and FA were measured using laser diffraction. Alcohol was used as the dispersant, and the obtained PSDs are presented in Fig. 2. It can be seen that the particles of NC1 and NC2 are finer than that of PC and FA. The average particle diameter of NC1 and NC2 is 2.13 mm and 3.03 mm, respectively, due to the agglomeration of NC to form micron particles, see Fig. 1(a,b). Figure 3 shows the XRD analysis of NC1, NC2, PC, and FA. Calcite peaks of NC2 are lower than that of NC1, which may be attributed to a small amount of phases in minority. These minorities are not visible in the XRD pattern but reducing the intensity of peaks of calcite. Crystal phase compositions of FA are mainly gypsum, mullite, and quartz. The presence of gypsum in FA can be attributed to the capture of SO_2_ in the carbon combustion tail gas in a power plant^27^. And the main constituent of the PC observed by XRD analysis is C_3_S.

### Methods

The mix proportions of specimens are shown in Table 2. The NC was mixed with PC, FA, and fine aggregate before adding tap water. Due to the high specific surface area of the NC, a HRWRA is used to maintain the fluidity of specimens. All specimens were stirred thoroughly in a mixer. The water to binder ratio (W/B) is 0.4. The specimens were cast into prismatic mold of size 40 × 40 × 160 mm, and demoulded after 24 hours. The specimens were then cured in saturated lime water at 20 ± 2 °C until the testing days. The strength of specimens was tested at 3, 7, 28, 60, 90, 180, and 360 days. SEM and XRD were used for microstructure evaluations at 3, 28, and 180 days. TG analysis and DTG analysis were used to calculate the CH content in specimens. DTG indicates the thermal decomposition of CH in the specimens, while TG measures the weight loss caused by CH decomposition simultaneously. Aproximitely 50 mg of powdered specimens were heated from ambient to 1000 °C (10 °C/min) in a nitrogen atmosphere. Specimens for TG/DTG analysis were obtained from 3, 28, and 180 days specimens.

**Table 2:** Mix proportion of the specimens.

Specimens	W/B	Cement/%	FA/%	NC1/%	NC2/%	HRWRA*/%	Sand to binder ratio
PCFA	0.4	60.0	40.0	—	—	—	3
NC1-2	0.4	58.8	39.2	2	—	0.5	3
NC1-4	0.4	57.6	38.4	4	—	0.6	3
NC1-6	0.4	56.4	37.6	6	—	0.7	3
NC1-8	0.4	55.2	36.8	8	—	0.8	3
NC2-2	0.4	58.8	39.2	—	2	0.5	3
NC2-4	0.4	57.6	38.4	—	4	0.6	3
NC2-6	0.4	56.4	37.6	—	6	0.7	3
NC2-8	0.4	55.2	36.8	—	8	0.8	3

*HRWRA dosages are added as percentage of binder.

## Results and Discussion

### Effects of NC on the strength of FA mortar

**Figure 4:**
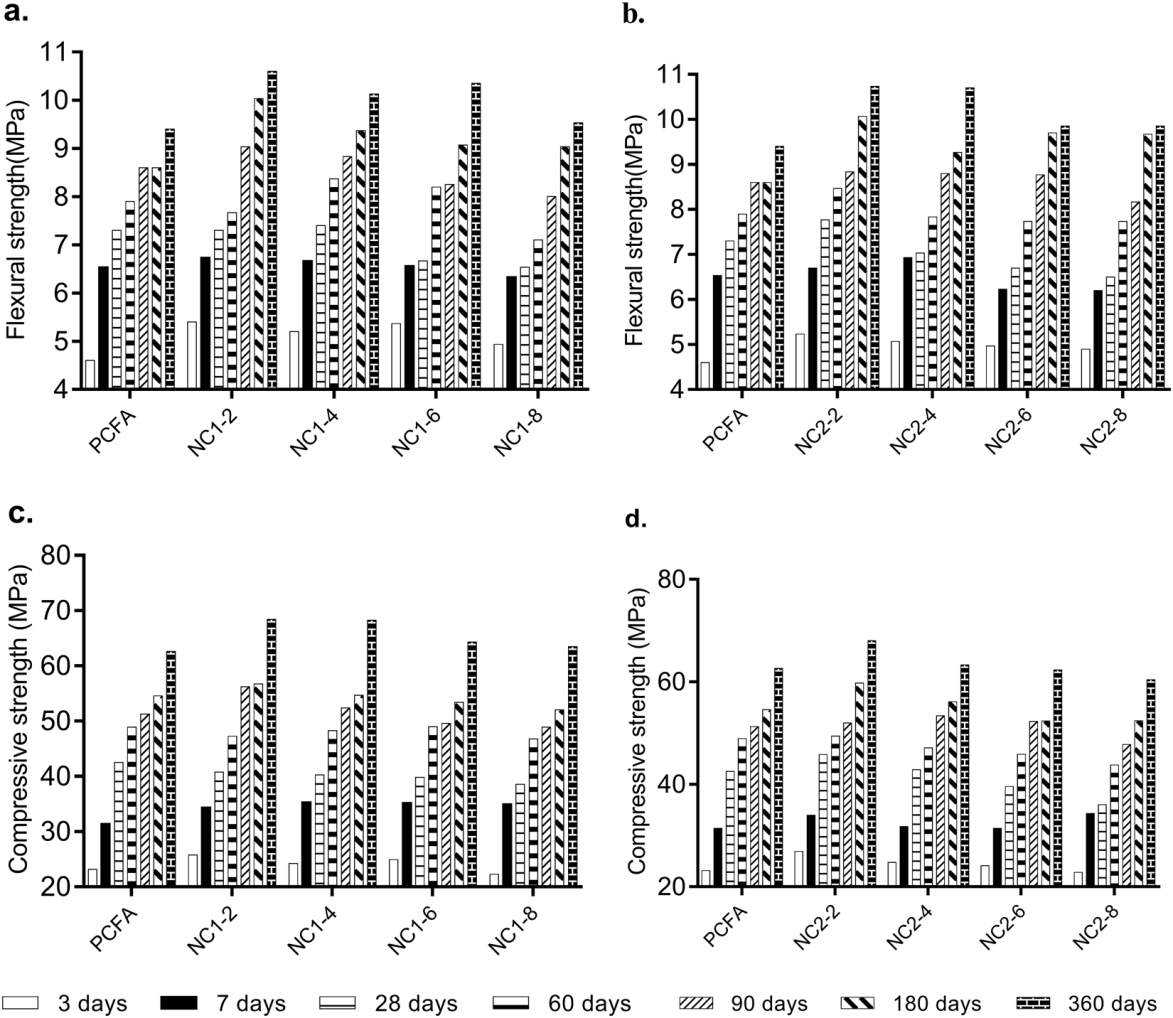
Flexural strength and compressive strength at different ages ((**a**) flexural strength of PCFA, NC1-2, NC1-4, NC1-6, and NC1-8; (**b**) flexural strength of PCFA, NC2-2, NC2-4, NC2-6, and NC2-8; (**c**) compressive strength of PCFA, NC1-2, NC1-4, NC1-6, and NC1-8; (**d**) compressive strength of PCFA, NC2-2, NC2-4, NC2-6, and NC2-8).

The effects of NC on the strength of FA mortar are presented in Fig. 4. As can be seen from Fig. 4, the speciman incorporating of 2% NC exhibit the highest flexural and compressive strength at 3 days. Then, the flexural and compressive strength is decreased gradually with the increase in NC contents. The lower compressive strength of NC1-8 can be attributed to the higher degree of agglomeration of NC1. Hence, about 2% NC is optimal incorporation, which contributes the most significantly to the strength of FA mortar. The NC2-2 exhibits of 13.0% and 15.9% higher flexural and compressive strength respectively than control mortar (PCFA) at 3 days, indicating the effectiveness of NC in offsetting the low strength at early age of FA mortar. Similar behavior is also observed in mortar with 2% or 4% NC at 7 and 28 days. However, the strength of FA mortar with NC2 was equal to or less than PCFA at 60 days. The lower strength of FA mortar containing higher NC can be attributed to the poor dispersion of nanoparticles^25^. With the increase of age, the strength of FA mortar with NC increased quickly. For instance, the compressive strength of PCFA at 90 days is 51.3 MPa, which gradually increases to 56.2 MPa when 2% of NC1 is added. Afterward, this value slightly decreases to 48.9 MPa when 8% of NC1 is included. Furthermore, it is noted that the specimen incorporating 2% of NC exhibited the highest strength at both 180 and 360 days, and the compressive strength are decreased with the increase in NC contents. For example, the NC2-2 mortar exhibit of 18.8% and 9.2% higher flexural and compressive strength respectively than PCFA at 180 days, indicating the effectiveness of NC in offsetting the low strength of FA mortar at long-term strength. Therefore, the optimal amount of NC is about 2%, which contributes the most to the long-term strength of the FA mortar.

**Figure 5:**
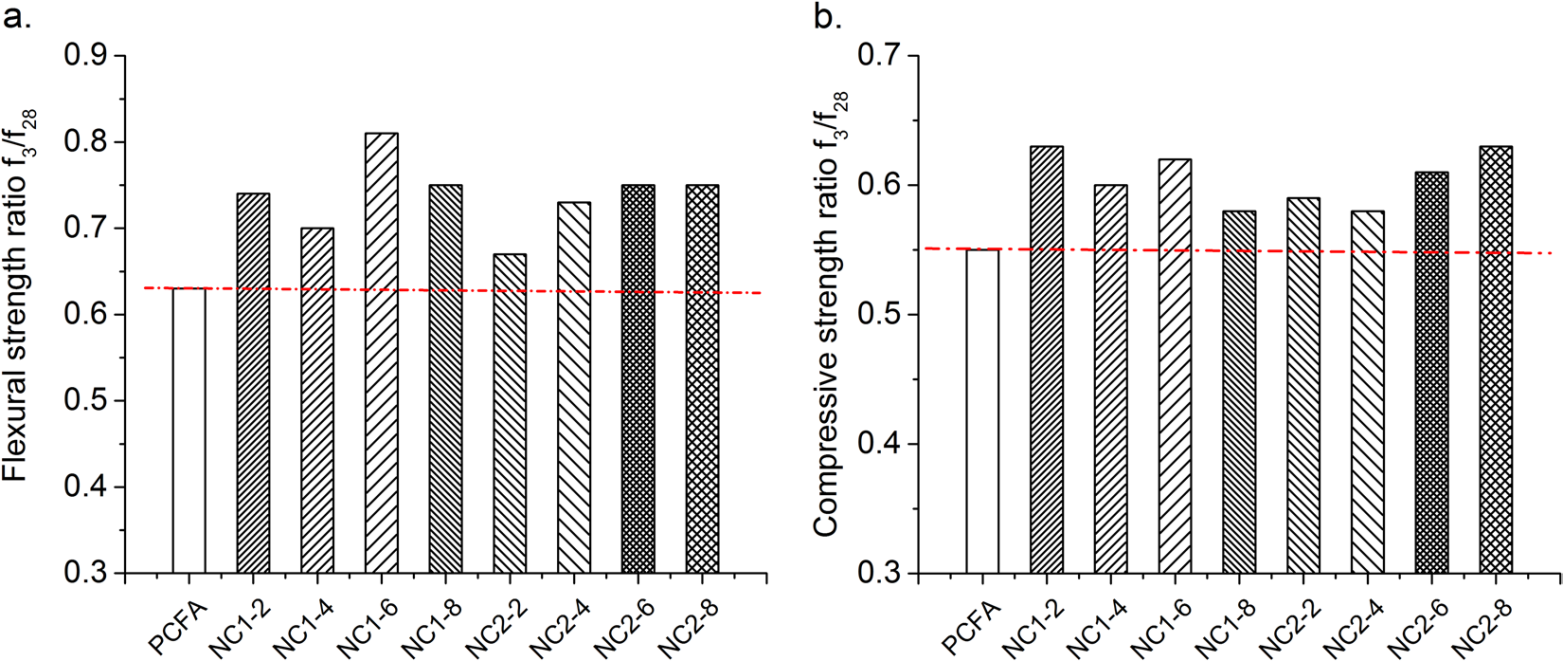
Strength ratio between 3 days and 28 days ((**a**) flexural strength ration between 3 days and 28 days; (**b**) compressive strength ration between 3 days and 28 days).

Figure 5(a,b) indicate the strength ratio between 3 and 28 days of FA mortar. Compared with PCFA, f_3_/f_28_ (strength ratio between 3 and 28 days of FA mortar) of specimens with NC increase dramatically. For instance, NC1-2 mortar exhibit about 17.5% and 14.5% higher flexural and compressive strength ratio, compared with PCFA. This may be due to accelerating effect and filling effect of NC at 3 days. Therefore, it is concluded that NC is helpful to increase the early-age strength of FA mortar.

### SEM analysis

**Figure 6:**
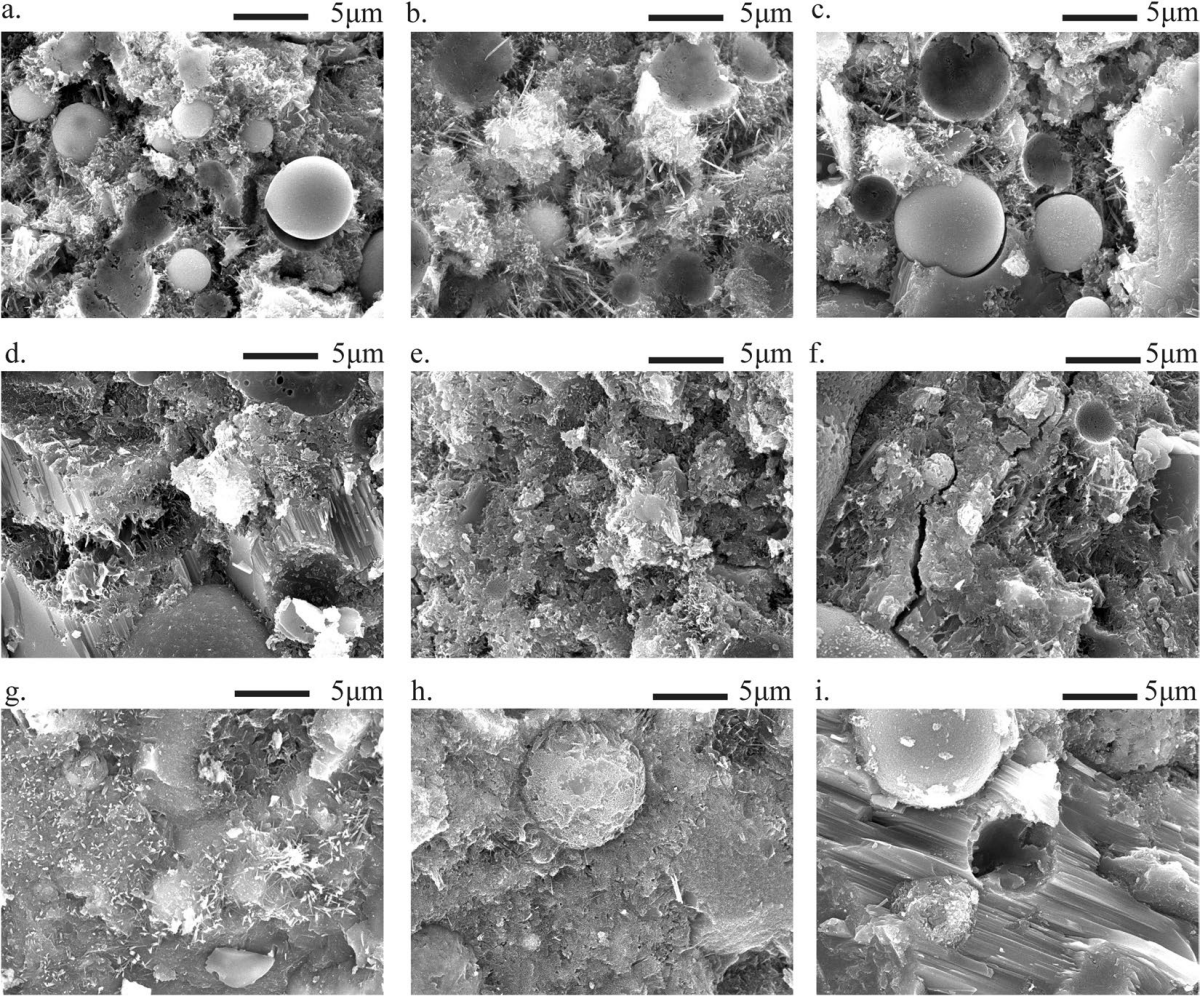
SEM micrographs of specimen ((**a**) PCFA hydrated at 3 days; (**b**) NC1-2 hydrated at 3 days; (**c**) NC2-2 hydrated at 3 days; (**d**) PCFA hydrated at 28 days; (**e**) NC1-2 hydrated at 28 days; (**f**) NC2-2 hydrated at 28 days; (**g**) PCFA hydrated at 180 days; (**h**) NC1-2 hydrated at 180 days; (**i**) NC2-2 hydrated at 180 days).

To better study the effects of NC on strength development of FA mortar, the morphology of hydrated products at 3, 28, and 180 days are examined through SEM. Figure 6(a–c) gives SEM images of FA mortar with and without NC at 3 days. From Fig. 6(a), a large amount of porous areas, calcium silicate hydrate (C-S-H), and AFt can be found in PCFA. However, due to the filling effect of nanoparticles, the dense microstructure of FA mortar incorporated with NC can be seen in Fig. 6(b,c). In addition, the NC accelerates the hydration of paste as they provide additional surface for the nucleation and growth of hydrated products^28^. The EDX analysis shows that the FA mortar with NC exhibits lower sulphate to alumina ratio than the control one. This observation suggests that calcium carbonate can react with FA containing high silica and alumina, thus increasing the strength of mortar^29,30^.

Figure 6(d–f) show the SEM images of FA mortar with and without NC at 28 days. It can be seen that the hydrated products are deposited on the FA particles and grow thicker with an ongoing hydration of paste. Furthermore, the FA would be gradually dissolved to form secondary C-S-H gel at late ages^31^, which refines the microstructure of FA mortar.

Figure 6(g–i) give SEM images of FA mortar with and without NC at 180 days. As can be seen from Fig. 6(g), large amount of acicular AFt, flake CH, spherical FA particles embedded in C-S-H gel can be observed in PCFA. However, as showed in Fig. 6(h,i), only a small amount of AFt was observed in the FA mortar with NC. This indicates that the NC reacts with aluminate, resulting in a more stable calcium carbonaluminate hydrate than AFt^32^. Furthermore, the microstructure of FA mortar with NC is denser than that of PCFA, which limits the growth of AFt.

Furthermore, it can be observed that the surface of FA particles is consumed due to the high alkalinity of hydrated products. This observation suggests that NC can react with FA containing high silica and alumina, therefore, influences the strength of mortar^29,33^. It is pointed out that the FA would be hydrated at late ages^31^, which generate more C-S-H gels, thereby increasing the compactness of FA mortar. More C-S-H gels on the surface of FA in NC1-2 and NC2-2 can be attributed to the accelerating effects of NC at 180 days. It is proposed that the additional surface provided by NC for the nucleation and growth of the hydrated products is the cause for the observed acceleration^20^.

**Figure 7:**
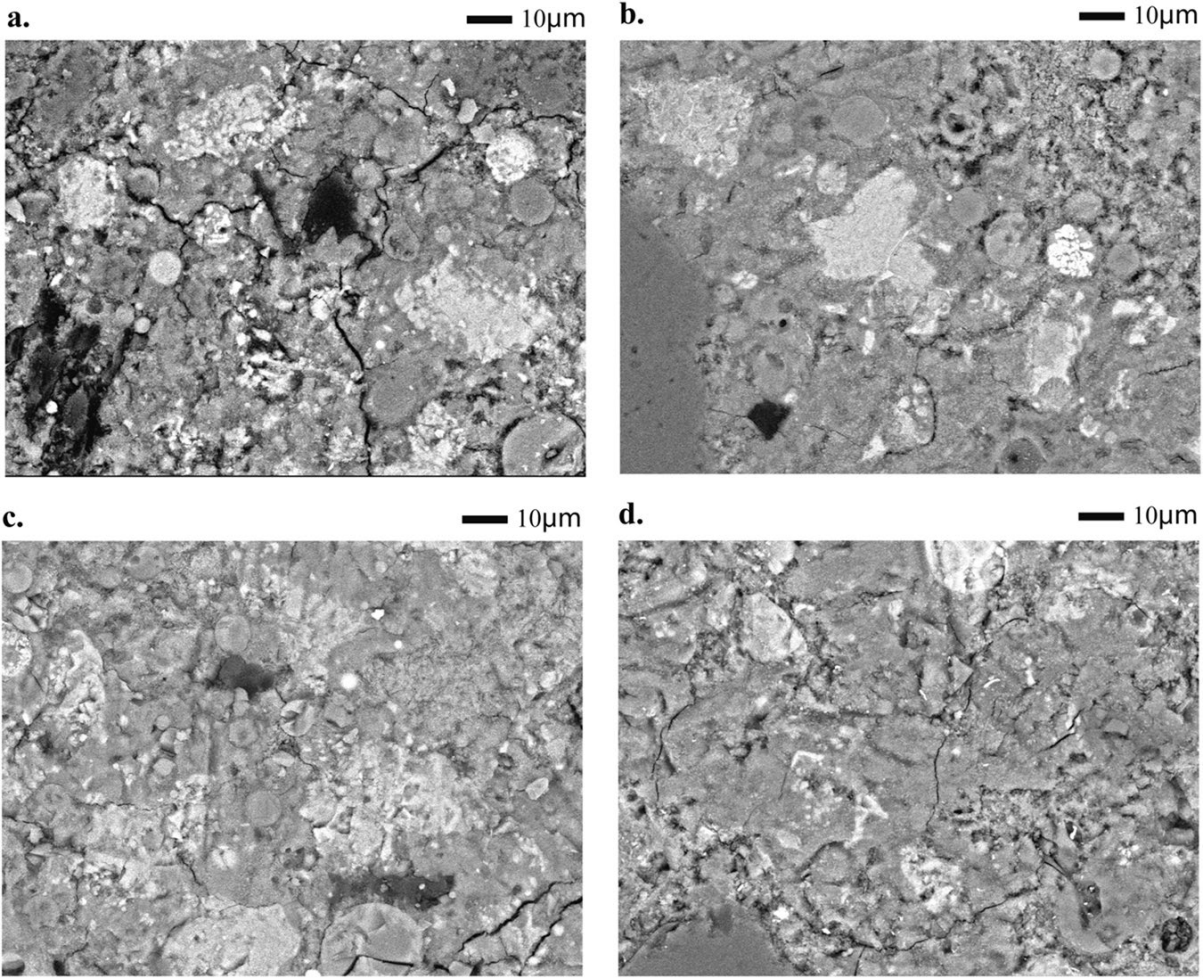
Backscattered electron images of specimen ((**a**) PCFA hydrated at 3 days; (**b**) PCFA hydrated at 28 days; (**c**) NC2-2 hydrated at 3 days; (**d**) NC2-2 hydrated at 28 days).

Backscattered electron images of specimens have also been used to investigate the microstructure changes of FA mortar. Figure 7(a–d) show the backscattered electron images of FA mortar with and without NC at 3 and 28 days. The constituent phase of the specimens can be identified by their brightness in the images. As can be seen, brighter areas correspond to partially reacted cement grains, followed by cement hydrates and finally the black spots as pores^34,35^. Compared to PCFA, NC2-2 has less white and black areas but more grey areas at 3 days, which indicate that NC2-2 has less partially reacted cement particles and pores but more hydrated products. This may be due to the seeding effect and filling effect of NC. Similar phenomenon is also observed in Fig. 7(b,d), which implies that the microstructure of NC2-2 is more dens than that of PCFA at 28 days. The result indicates that the incorporation of NC effectively refines pores by performing seeding and filling role, and thus densify the microstructure of FA mortar.

### XRD analyses

**Figure 8:**
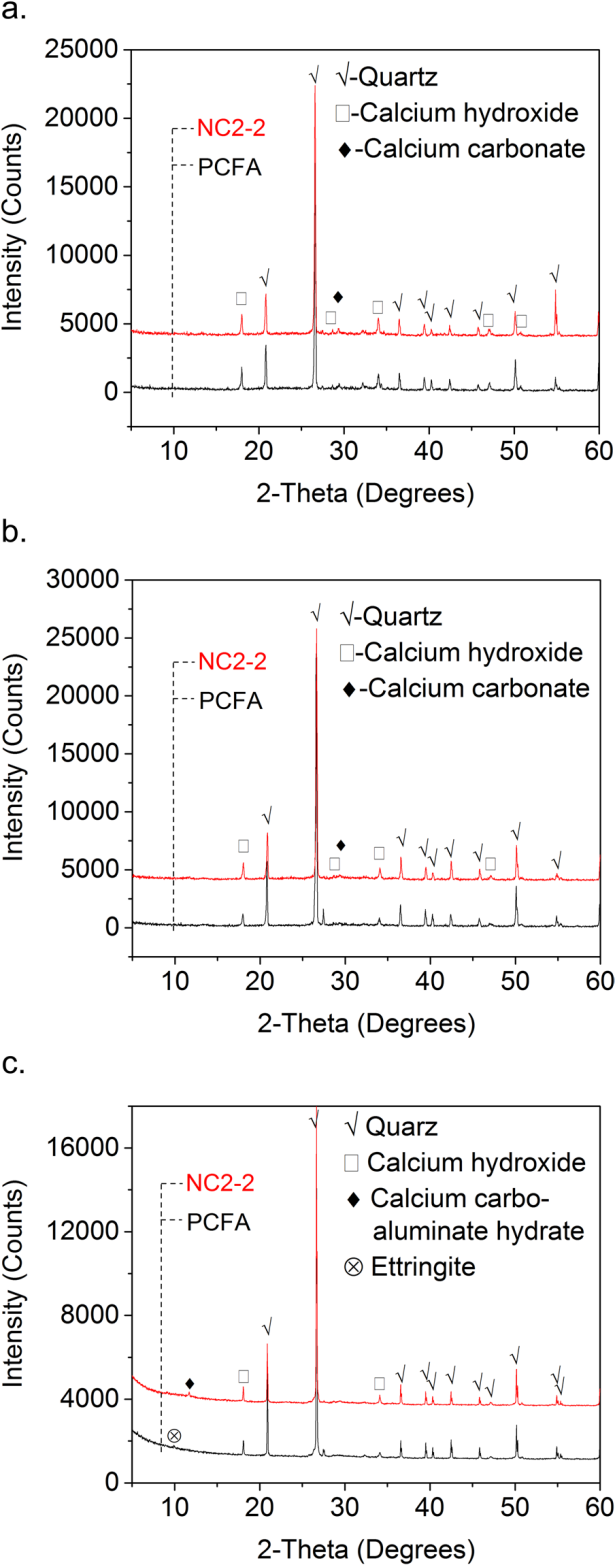
XRD pattern of specimens ((**a**) hydrated at 3 days; (**b**) hydrated at 28 days; (**c**) hydrated at 180 days).

The observation of SEM is further validated through XRD analysis. Figure 8(a–c) show the XRD patterns of FA mortar with and without NC at 3, 28, and 180 days respectively, indicating the predominance phase of the specimen is CH, calcium carbonate, and Quartz.

It is recognized that the strength of cementitious materials primarily depends on C-S-H. However, it is difficult to detect the C-S-H in XRD analysis. Therefore, the diffraction peak of CH is usually used to indirectly indicate the amount of hydrated products. The diffraction spectra of PCFA and FA mortar containing 2% NC2 shows that the CH peak intensity in NC2-2 is lower than that in PCFA at 3 days, while at 28 days, the CH peak intensity in NC2-2 is higher than that in PCFA. For instance, the CH intensity decreased from 1644 to 1481 at 2θ = 18.04° after 3 days, while, the CH intensity increased from 984 to 1401 at 2θ = 18.04° after 28 days. The decrease in CH diffraction peak may be due to the pozzolanic reaction of CH with FA to form additional hydrated products. In addition, calcium carbonate reacts with aluminate phases in cement, which deplete CH^32^. Both calcium hemicarboaluminate hydroxide and calcium monocarboaluminate hydroxide were not detected. The increase in CH content is correlated with the acceleration effect of NC2. The NC performs as nucleation surface for hydrated products, such as CH and C-S-H^20^. Pera^22^ and Kakali^36^ also believe that calcium carbonate could react with C_3_S and accelerate the hydration of C_3_S, which increase the production of CH. The increase of CH contributes to the strength development of FA mortar containing NC. At 180 days, diffraction spectra analysis in PCFA indicates the predominance of quartz, CH and AFt. The diffraction peak of AFt did not appear in the FA mortar with NC, but the diffraction peak of calcium carbonaluminate hydrate appeared at 2θ = 11.7°. This is due to the reaction of NC react with aluminate, resulting in a more stable calcium carbonaluminate hydrates than AFt. The CH peak intensity in NC2-2 is higher than that in PCFA at 2θ = 34.1° after 180 days. This increase in CH is correlated to the acceleration effect of NC. The NC performs as nucleation surface for the hydrated products^20^.

### TG/DTG analyses

**Figure 9:**
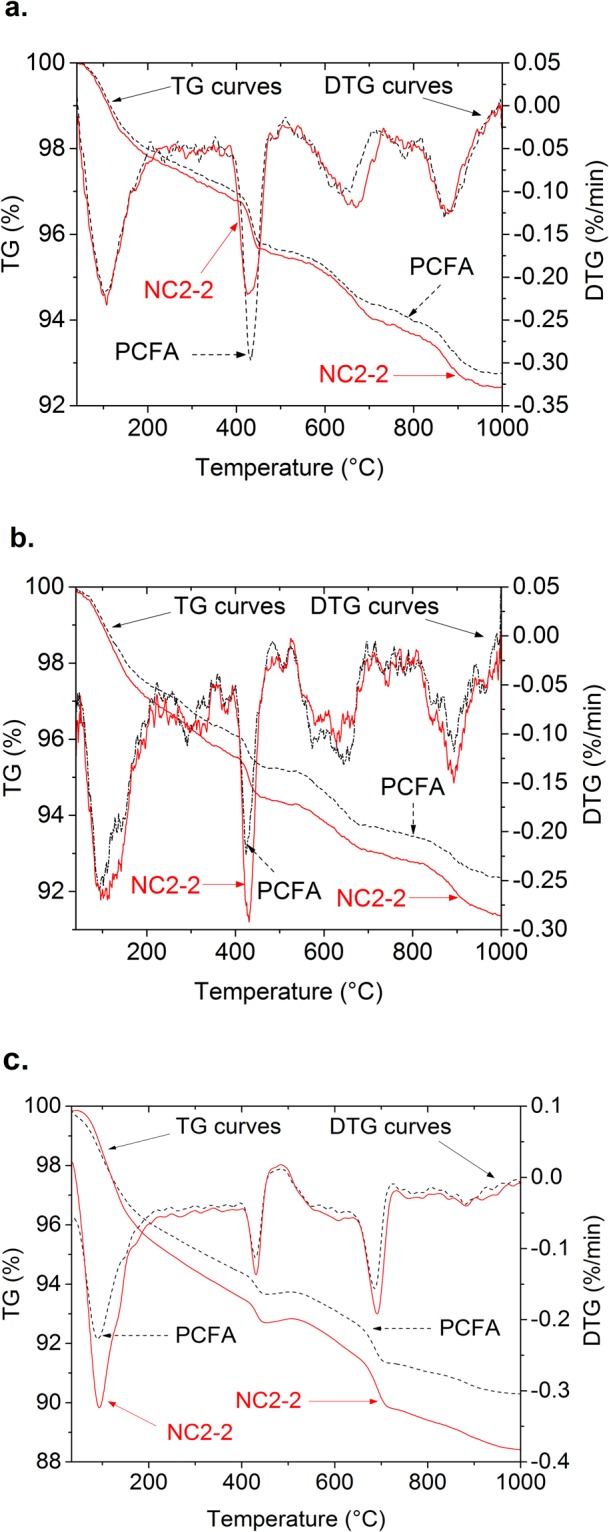
TG-DTG digrams ((**a**) hydrated at 3 days; (**b**) hydrated at 28 days; (**c**) hydrated at 180 days).

To analyse the effects of NC on the hydrated products of FA mortar, control specimen and specimen containing 2% of NC at 7, 28, and 180 days were conducted the TG/DTG analysis test. The TG and DTG results of PCFA and NC2-2 are presented in Fig. 9. The DTG spectrum is used to identify the detected phase of the specimens, while the TG spectra shows the normalized mass loss in percent. It can be observed that the TG spectras show three major mass losses: (1) dehydration of hydrates (C-S-H, C-A-H, AFt and AFm phases), in the range of 105 to 200 °C; (2) dehydroxylation of CH about 430 °C; and (3) decomposition of CaCO_3_ above 550 °C. TG curves show the mass loss of specimens heated from ambient temperature to about 1000 °C. Non-evaperable water and CH thus can be calculated from Fig. 9. Results are listed in Table 3.

**Table 3:** CH content and bound water content.

	CH content (%)	Bound water content (%)
PCFA		
3 days	5.5	1.7
28 days	3.6	2.3
180 days	3.2	3.6
NC2-2		
3 days	5.3	1.8
28 days	4.8	2.6
180 days	3.3	4.5

As shown in Fig. 9 and Table 3, the bound water content of all specimens increases with curing ages, but the CH content of all specimens decreases with curing ages. The amount of bond water in hardened cementitious materials is approximately proportional to the degree of hydration. Therefore, the increase of bound water with curing ages indicates the increase of hydration degree of cement. This phenomenon is consistent with the strength development shown in Fig. 4. The decrease of CH content of specimens with curing ages indicates that FA react with CH to form additional C-S-H and C-A-H. These hydrated products improved the strength of FA mortar.

It also can be seen from Fig. 9 and Table 3 that CH contents of FA mortar containing 2% NC2 are lower than that of PCFA at the age of 3 days, while bound water content of NC2-2 is higher than that of PCFA. This could be due to the reaction between NC and aluminate phases, which deplete CH. At 28 days, the CH content and bound water content in NC2-2 are higher than that of PCFA, indicating that NC accelerates the hydration reaction of cement and thus produces more CH. At 180 days, non-evaperable water and CH contents of FA mortar with 2% NC are higher than that of PCFA, indicating that NC accelerates the hydration of cement. Therefore, NC has a great effect on the hydrated products of FA mortar. The TG/DTG results explain the difference in mechanical performances between FA mortar with and without NC. The similar results were also obtained in the XRD analysis as discussed above.

## Conclusions

This work studied the effects of NC on the strength development and microstructure of FA mortar. It can be concluded from the experiments in this study that:

1. Incorporating NC has positive effects on the strength of FA mortar not only at early ages but also at later ages.
2. The highest strength occurs in the FA mortar incorporating about 2% NC2 at all curing ages. Higher dosage of NC1 (i.e. >6%) will decrease the strength achieved, which can be attributed to the dilution effect.
3. From the point of view of strength development, adding about 2% NC can effectively improve the early-age mechanical performances of FA mortar, thereby compensating for the retarding effect of FA. Also, NC is much cheaper than silica nanoparticles.
4. The hydration reaction of cement was accelerated when the NC is added to FA mortar. The additional nucleation sites provided by NC induced the more rapid growth of hydrated products is the key contributors to the improved strength of FA mortar.
5. Test results of SEM show that the presence of 2% NC2 improved the microstructure of FA mortar thus improve the flexural and compressive strength. The improvement is further confirmed by backscattered electron image analysis that 2% NC2 reduce the pore size and porosity inside the FA mortar.
6. Test results of XRD show that the NC reduces the intensity of CH at 3 days and increases the intensity of CH at 28 and 180 days in FA mortar. The decrease in CH intensity due to the reaction of CH with FA containing high silicate and aluminate to form additional hydrated products, therefore, contributes to the improvement of strength of FA mortar at 3 days. While, the increase in CH intensity at 28 and 180 days is correlated with the acceleration effect of NC. The TG-DTG results are further confirmed the XRD results.
7. Test results of SEM and XRD show that the presence of 2% NC improved the microstructure of FA mortar after 180 days due to the formation of calcium carbonaluminate hydrate and the acceleration effects of NC, thus improve the flexural and compressive strength. The improvement is further confirmed by DTG/TG analysis.

## Data Availability

All data included in this study are available upon request by contact with the corresponding author.
